# Arterial phase enhancement and body mass index are predictors of response to chemoembolisation for liver metastases of endocrine tumours

**DOI:** 10.1038/sj.bjc.6603526

**Published:** 2006-12-12

**Authors:** F Marrache, M P Vullierme, C Roy, Y El Assoued, A Couvelard, D O'Toole, E Mitry, O Hentic, P Hammel, P Lévy, P Ravaud, P Rougier, P Ruszniewski

**Affiliations:** 1 Service de Gastroentérologie, hôpital Beaujon, AP-HP, Université Paris 7, Clichy, France; 2 Service de Radiologie, hôpital Beaujon AP-HP, Clichy, France; 3 Département d’Epidémiologie, Biostatistique et Recherche Clinique, Groupe Hospitalier Bichat-Claude Bernard, AP-HP – Université Paris 7, Clichy, France; 4 Service d’Hépato-Gastroentérologie, hôpital Ambroise Paré, AP-HP, Clichy, France; 5 Service d’Anatomie et Cytologie Pathologiques, hôpital Beaujon, AP-HP, Clichy, France

**Keywords:** chemoembolisation, endocrine tumour, streptozotocin, body mass index, tumour vascularisation

## Abstract

Transcatheter arterial chemoembolisation (TACE) has been reported to be an efficient treatment of liver metastases of endocrine tumours in short series of patients. However, several factors seem to affect its results. The aim of this work is to identify predictors of response to TACE for liver metastases of endocrine tumours. A total of 163 TACE procedures were performed in 67 patients between 1994 and 2004. Forty-four patients were treated with streptozotocin and 23 with doxorubicin. Primary tumour was located in the pancreas for 19 patients, and had been removed in 43. Thirty-eight tumours were functioning. Response rate was 37% (confidence interval [CI] 95%: 28–49%). Median time to progression (TTP) was 14.5 months (CI 95%: 9–41). In multivariate analysis (*n*=43), predictors of tumour response were body mass index (BMI) (odds ratio [OR]: 1.3; CI 95%: 1.04–1.63; *P*=0.022), functioning type of tumour (OR: 7.31; CI 95%: 1.26–42.5; *P*=0.027), arterial phase enhancement on abdominal computed tomography (CT) (OR: 8.11; CI 95%:1.06–62; *P*=0.044) and use of streptozotocin for cytotoxic agent (OR: 21.3; CI 95%: 1.48–306; *P*=0.025). Analysis of TTP predictors showed that BMI (hazard ratio [HR]: 0.85; CI 95%: 0.76–0.86; *P*=0.01) and arterial phase enhancement (HR: 0.3; CI 95%: 0.12–0.73; *P*=0.008) were associated with delayed progression. This large study confirms the previously reported results of TACE regarding its efficacy for the treatment of liver metastases of endocrine tumours. Arterial phase enhancement on abdominal CT and BMI are predictors of treatment's efficacy. Streptozotocin should be the preferred cytotoxic agent in order to save anthracycline for systemic chemotherapy.

Endocrine tumours are rare tumours of the digestive tract (Modlin *et al*, 2003; Warner, 2005). Their main feature is the secretion, by some of them, of biologically active substances or hormones responsible for typical syndromes and requiring a specific management. Their prognosis is usually considered as being favourable because of their slow-growing pattern, even though this needs to be discussed according to several factors affecting patients’ survival. Among these, the presence of liver metastases is one of the most important (Madeira *et al*, 1998; Gullo *et al*, 2003; Modlin *et al*, 2003; Plockinger *et al*, 2004).

First-line treatment is then surgery, as it is the only treatment that allows a definite cure. However, in case of extensive metastases, complete resection might be precluded. The aim of therapy is then to control hormone-related symptoms and tumour growth using different available approaches: somatostatin analogues, interferon alpha, intravenous chemotherapy, peptide receptor-targeted radiotherapy, vascular occlusive therapy and transcatheter arterial chemoembolisation (TACE) (Mitry *et al*, 1999; Plockinger *et al*, 2004).

The rationale of TACE is to combine the effects of chemotherapy injected in the hepatic artery, with those of anoxia induced by hepatic artery embolisation (Ruszniewski and O’Toole, 2004). Indeed, certain drugs are more efficient on anoxic cells (Roche, 1989) and reduced vascularisation can increase intratumoral drug concentration and dwelling time in cancer cells (Bengmark *et al*, 1974; Delaunoit *et al*, 2004).

Transcatheter arterial chemoembolisation is effective to control hormone-related symptoms in 70–100% of patients, especially in those with carcinoid syndrome. Tumour response rate has widely varied between 9 and 60% (Ruszniewski *et al*, 1993; Therasse *et al*, 1993; Diaco *et al*, 1995; Dominguez *et al*, 2000; Gupta *et al*, 2003; Kress *et al*, 2003; O’Toole *et al*, 2003; Roche *et al*, 2003, 2004), partly explained by the limited case numbers (most studies involve less than 35 patients) and presumably by some factors related to the patients, their tumours, TACE protocol and previous treatments.

The aim of this study was to identify predictors of response to TACE for liver metastases of endocrine tumours in a large series of patients.

## PATIENTS AND METHODS

### Patients

We conducted a retrospective bicentric study. Every patient with liver metastases of endocrine tumours referred to Beaujon (*n*=67) or Ambroise Paré (*n*=13) Hospitals for TACE between 1994 and 2004 was recorded. Among these 80 patients, 67 were included in our study. The remaining 13 patients were excluded because the radiological documents were not available for re-evaluation. Baseline clinical and radiological features listed in Table 1 were recorded.

Concomitant use of somatostatin analogues was recorded when patients were receiving long-term treatment, but not in case of periprocedural administration. Indication for treatment was a progressive disease before TACE, determined on two consecutives abdominal computed tomography (CT) or magnetic resonance imaging (MRI) procedures and confirmed on re-evaluation by an experienced radiologist (MPV) according to RECIST criteria (Therasse *et al*, 2000) or uncontrolled hormone-related symptoms or an aggressive disease defined by extensive metastases (>50% replacement of liver parenchyma) and tumour-related symptoms.

Assessment of baseline radiological features relied on re-evaluation by an experienced radiologist (MPV) blinded to treatment outcome. In Beaujon Hospital, multiphasic helical CT was performed with two consecutive CT machines. First, a CT Twin Marconi (Haifa, Israel) was used (*n*=42): unenhanced phase (section thickness 5 mm) was followed by enhanced study at late arterial phase referred to as the pancreatic phase (section thickness 2.5 mm, pitch 1.5) and during the portal venous phase (section thickness 5 mm, pitch 1.5) with a delay of 45 and 70 s, respectively, after initiation of the intravenous bolus injection of 100–120 cc of low osmolality contrast material iodine at a flow rate of 3 cc/s. Ingestion of water was obtained. During a second period, a General Electric CT Light Speed Ultra (GE Healthcare, Milwaukee, WI, USA) was used (*n*=13): acquisition was performed with 1.25 section thickness. Reconstruction during the unenhanced phase (section thickness 5 mm, pitch 1.35), pancreatic phase (section thickness 2.5 mm, pitch 0.875) and the portal venous phase (section thickness 5 mm, pitch 0.875) were performed with a delay of 45 and 70 s, respectively, after initiation of the intravenous bolus injection of 100–120 cc of low osmolality contrast material iodine at a flow rate of 3 cc/s. Ingestion of water was obtained. In the second institution (Ambroise Pare Hospital), a Twin Flash scanner (Elscint, Haifa, Israel) was used (*n*=6). Unenhanced phase (section thickness 5 mm) was followed by enhanced study at late arterial phase referred to as the pancreatic phase (section thickness 2.5 mm, pitch 1.5) and during the portal venous phase (section thickness 5 mm, pitch 1.5) with a delay of 45 and 70 s, respectively, after initiation of the intravenous bolus injection of 100–120 cc of low osmolality contrast material iodine at a flow rate of 3 cc/s.

The percentage of liver replacement was semiquantitatively quoted (<25, 25–50, 50–75 and >75%). Arterial phase enhancement was defined on the arterial phase images (when available) of the pretreatment CT as a hyperattenuation of the lesion compared to the adjacent liver parenchyma. No measure of the density was performed. A heterogeneous pattern of the metastases was defined as the presence of variously attenuating areas in a single lesion, suggestive of necrosis, either before and/or after contrast medium enhancement (arterial and/or portal phase).

### Treatment

Intravenous hydration was started on 3 h before TACE and patients were premedicated with antibiotics. Cytotoxic drugs were doxorubicin (50 mg m^−2^) or streptozotocin (1.5 g m^−2^). The latter was chosen in case of pancreatic primary tumour or contraindication to anthracycline use. Patients with carcinoid tumours were administered octreotide 200 *μ*g subcutaneously twice daily for 10 days beginning 24 h before TACE to prevent carcinoid crisis. A diagnostic superior mesenteric and celiac trunk arteriography was first obtained to evaluate the distribution of hepatic arteries and portal blood flow. Cytotoxic drug dissolved in normal saline was combined with 10 ml of iodized oil, and injected into the branches of the hepatic artery distal to the gastroduodenal artery. This was followed by embolisation with gelatin sponge particles until a marked decrease in blood flow was observed. As injection of streptozotocin containing mixtures has been reported to be painful (Dominguez *et al*, 2000), probably in relation with its acidity, procedures were then performed under general anaesthesia.

Treatment was repeated every 3 months. In case of objective response or stable disease, usually after two procedures had been performed, treatment could be temporarily interrupted until symptomatic relapse or tumour progression.

### Evaluation of tumour response

Follow-up evaluations were performed every 3 months. They were based on clinical evaluation, tumour markers measurements when available (Chromogranin A or specific marker) and tumour response assessment on CT or MRI. Originals CT or MRI films were re-evaluated by an experienced radiologist (MPV). The primary criterion of tumour response evaluation in this study was that defined according the Response Evaluation Criteria in Solid Tumors (RECIST) (Therasse *et al*, 2000): Complete response (CR) was defined as the complete disappearance of all lesions; partial response (PR) was defined as at least a 30% reduction in the sum of the longest diameters of up to five target lesions, taking as a reference the baseline sum of the longest diameters; progressive disease (PD) was defined as at least a 20% increase in the sum of the longest diameters or development of new lesions or global deterioration of health status requiring treatment's discontinuation. Stable disease (SD) was defined as disease that showed neither sufficient shrinkage nor increase to qualify as either PR or PD.

Date of progression was defined as the date criteria for PD are met, taking as a reference the smallest measurements recorded since treatment started. For patients whose treatment was temporarily suspended, who had several periods of treatments, date of progression was the date of the last evaluation demonstrating PD before treatment's failure. Duration of overall response was defined as the time between the initial documented response and the first date of recurrence or progression.

Clinical response evaluation could be retrospectively performed in 23 patients and was based on the report of hormone-related symptoms and symptomatic treatment in patients’ file. Complete clinical response was defined as complete disappearance of all hormone-related symptoms without any symptomatic treatment; partial clinical response was defined as an improvement of symptoms without any increase in symptomatic treatment or a decrease of this treatment without any increase of symptoms. Clinical progression was defined as an increase in hormone-related symptoms or in symptomatic treatment. Clinically stable disease was defined by the absence of modification of patient's symptoms and symptomatic treatment.

Biological response was evaluated in 38 patients based on plasmatic chromogranin A level or any tumour-type-specific marker when appropriate. Complete biological response was defined as a complete normalisation of all tumour markers and partial biological response was defined as a decrease of more than 50% compared to baseline level. Biological progression was defined as an increase of 25% of any tumour marker and in case tumour markers had neither increase or decrease sufficiently to demonstrate biological progression or response, disease was biologically stable.

When available, MIB-1 quantification performed either on primary tumour or liver metastases was recorded from patients’ files.

Adverse effects related to the treatment were recorded from patients’ files.

### Statistics

The comparisons between tumour response according to RECIST criteria and baseline characteristics of the patients were based on *χ*^2^ and Fisher's exact tests for qualitative variables, and on an ANOVA for quantitative variables. Multivariate logistic regression was then performed in order to identify the predictors of response to treatment. A stepwise procedure with variables having a *P*-value less than 0.25 in univariate analysis was used.

Time to progression (TTP) was measured from beginning of treatment to date of progression, death, last follow-up, curative surgery or liver transplantation. Overall survival (OS) was measured from beginning of treatment to death or time of last follow-up. The survival function was calculated with the method of Kaplan–Meier. The differences of TTP according to baseline characteristics were analysed using the log-rank test. Multivariate analysis including variables with a *P*-value less than 0.25 was subsequently performed using a Cox proportional hazards model with a stepwise procedure. Differences were considered statistically significant if the *P*-value was less than 0.05. All analyses were performed using SAS version 9 (SAS Institute Inc., Cary, NC, USA).

## RESULTS

### Baseline characteristics

Baseline clinical and radiological features of the patients are shown in Table 1. Transcatheter arterial chemoembolisation was prompted by a progressive disease for 41 patients, by uncontrolled hormone-related symptoms for 17 patients and by an aggressive disease for the nine remaining ones. The primary lesion was located in the small bowel (*n*=24), the pancreas (*n*=19), the rectum (*n*=4), the duodenum (*n*=3), the appendix (*n*=3), the bronchus (*n*=3), the stomach (*n*=2), the kidney (*n*=1) and the large bowel (*n*=1). In seven cases, no primary lesion was found. Thirty-eight tumours were functioning. These were carcinoid tumours (*n*=31), gastrinomas (*n*=2), VIPomas (*n*=2), a somatostatinoma, a calcitonin-secreting tumour and a PTHrp-secreting tumour. Among these patients with functional tumours, 25 were treated with long-term somatostatin analogues. The other patients had only minor and infrequent symptoms that did not justify such therapy or tumour-type for which it was not relevant. Four patients had poorly differentiated tumours. Liver metastases were synchronous to the primary tumour in 58 patients. Twenty-six patients had extra-hepatic lesions corresponding to the unresected primary tumour and/or abdominal metastatic lymph nodes in 13 of them. Fifteen patients had previously undergone surgery for liver metastases and two pancreaticoduodenectomy. One patient had multiple endocrine neoplasia type 1. Transcatheter arterial chemoembolisation was performed with streptozotocin in 44 patients, including 25 with non-pancreatic primary tumour, but who received previously anthracycline chemotherapy (*n*=10) or had cardiac contra-indication to anthracycline (*n*=15) among which seven had severe carcinoid heart disease, requiring valvular replacement in three cases.

### Procedures

One hundred and sixty-three TACE procedures were performed during 168 attempts. Median number of procedures per patient was two, ranging from 1 to 8. Both hepatic lobes were treated in 138 cases, the right hepatic lobe in 20 cases and the left hepatic lobe in three cases. Two procedures were performed without any embolisation in relation with the presence of arterio-venous shunts.

### Response to treatment

**Figure 1:**
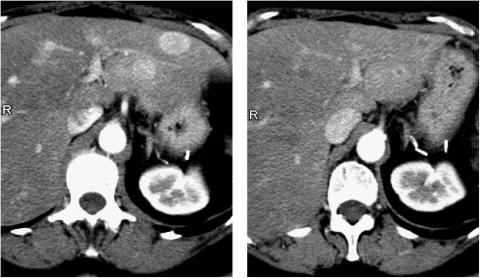
Liver metastases of endocrine tumour showing a hypervascular pattern before treatment (left panel) that shrinked after chemoembolisation (right panel).

Complete or partial tumour response according to RECIST criteria was observed in 37% (CI 95%: 28-49) of patients and 73% (CI 95%: 63–84) had CR, PR or SD, that is, a nonprogressive disease. The median duration of overall response was 27 months (CI 95%: 12–67). Median TTP was 14.5 months (CI 95%: 9–41). Clinical response was evaluated in 23 patients: 91% (CI 95%: 80–100) responded, completely for 61% (CI 95%: 41–81). Complete results about tumour, clinical and biological responses are shown in Table 2 and Figure 1.

### Predictors of tumour response

Identification of predictors of response to treatment was performed by two means: by studying variables associated with tumour response (complete or partial), or with TTP. Univariate analysis of variables listed in Table 3 was performed. Variables with a *P*-value <0.25 in these analyses were included in our multivariate models, except MIB-1 for which too many data were unavailable.

**Figure 2:**
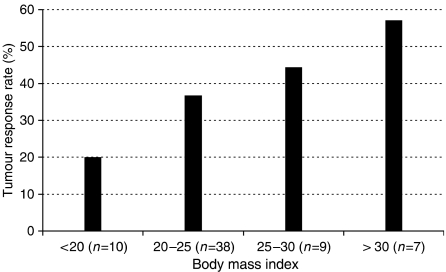
Tumour response rate according to BMI categories.

Multivariate logistic regression analysis of predictors of tumour response was performed in 43 patients. Body mass index (BMI) (odds ratio: 1.3; CI 95%: 1.04–1.63; *P*=0.022), functioning tumour (odds ratio (OR): 7.31; confidence interval (CI) 95%: 1.26–42.5; *P*=0.027), arterial phase enhancement on computed tomography (CT) (OR: 8.11; CI 95%: 1.06–62; *P*=0.044) and use of streptozotocin for cytotoxic agent (OR: 21.3; CI 95%: 1.48–306; *P*=0.025) were predictors of tumour response. There was no significant interaction between these variables. Additionally, we tested the following interactions: type of cytotoxic agent/primary location, functioning type of tumour/primary location, BMI/percentage of liver replacement, hypothesising a low BMI reflected an advanced disease, and somatostatin analogues treatment with all four of these identified factors. None of these interactions was significant in our model. Figure 2 shows the tumour response rate according to BMI category in our patients.

**Figure 3:**
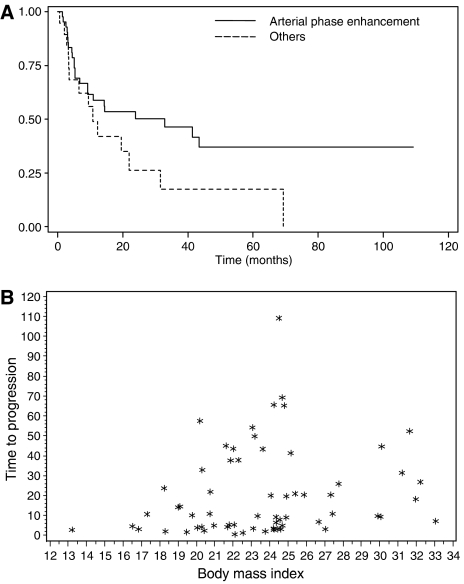
Predictors of time to progression (TTP). (**A**) Time to progression according to the presence of arterial phase enhancement on CT. (**B**) Distribution of TTP according to BMI.

In the Cox proportional hazards model of variables associated with TTP, 43 patients were included. Body mass index (HR: 0.85; CI 95%: 0.76–0.86; *P*=0.01) and arterial phase enhancement on CT (HR: 0.3; CI 95%: 0.12–0.73; *P*=0.008) were associated with delayed progression (Figures 1 and 3). Interactions between these variables, between each of them and somatostatin analogues treatment, and between BMI and percentage of liver replacement were not significant in this model.

### Side effects

Side effects are listed in Table 4. No treatment-related death was reported. The most common side effect was the post embolisation syndrome, associating vomiting, abdominal pain and fever. One patient had a liver abscess, whereas he had no previous story of biliary anastomosis or portal vein obstruction. Three episodes of acute liver failure followed treatment in two patients without carcinoid heart disease or portal vein thrombosis, and whose percentage of liver replacement was <50% and <75%, respectively. Each time, recovery was complete. Five carcinoid crises occurred in two patients, controlled with intravenous somatostatin analogues administration.

### Survival

**Figure 4:**
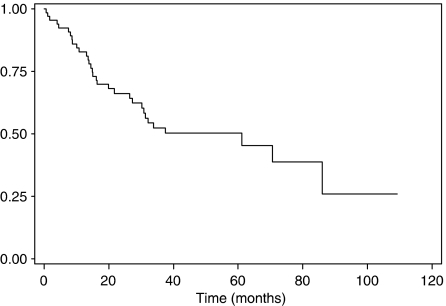
Overall survival.

Median overall survival after beginning of treatment was 61 months (CI 95%: 30–86), and overall survival rates at 2 and 5 years were 66 and 50%, respectively (Figure 4).

## DISCUSSION

With a tumour response rate of 37% (CI 95%: 28–49%), our results are in the range of previous studies regarding treatment's efficacy (Ruszniewski *et al*, 1993; Therasse *et al*, 1993; Diaco *et al*, 1995; Dominguez *et al*, 2000; Gupta *et al*, 2003; Kress *et al*, 2003; O’Toole *et al*, 2003; Roche *et al*, 2003). Recently, Roche *et al* (2004) reported a response rate of 74%, much higher than what we observed. However, in this study, minor and objective responses according to World Health Organization criteria for tumour response were aggregated. Objective response rate, which correlates with definition of response according RECIST criteria, was 40%, close to our findings.

A major pitfall of every clinical trial about endocrine tumours is the lack of information regarding tumour progression, making end points such as disease control or time to progression questionable. In our series, 41 patients had documented progressive disease so that it could be suggested that the remaining ones did not have a progressive disease. To clarify this issue, it must be pointed that among the remaining ones, nine had an aggressive disease defined by ⩾50% replacement of liver parenchyma. In our univariate analysis of factors associated with time to progression, this latter criterion appeared to be associated with an earlier progression (*P*=0.033, Table 3) and opposes the assumption that these patients had a stable disease even without TACE. Regarding the 17 other patients, their disease was not documented as progressive and it is thus right to point that disease control might be a debatable end point. This is the reason why we designed our study of predictive factors based on two different end points. The first one was tumour response (thus excluding stable disease) based on radiological criteria, and the second one was TTP.

Although the number of patients was somewhat limited, two factors were found significant in both analyses: arterial phase enhancement and BMI. As TTP could be an end point related to the natural history of the disease, it could be hypothesised that factors linked with it were in fact associated with the natural history of the disease. However, these were also predictive of radiological response, suggesting that any effect on time-related end points would be at least partly related to a treatment effect. A recent study by our group showed that microvessel density in pancreatic endocrine tumours is correlated with the vascular pattern of the tumour on helical CT (Rodallec *et al*, 2005). Knowing this, it appears consistent that the more vascularised the tumour, the better it responds to TACE. Although the CT were performed on different machines in the two institutions, the protocol of vascular opacification and the timing of images acquisition remained the same, and this should not constitute a significant bias in our study.

The other significant factor in both analyses was BMI. An explanation could be that patients with a low BMI do not tolerate treatment, although we did not find any evidence for the latter hypothesis, or are those whose disease is the most aggressive. The ECOG performance status might reflect tumour's aggressiveness, but statistical analysis could not be performed regarding this factor as most of the patients were in good general condition, so that only five had a performance status of 2. In addition, there was no significant interaction in our model between BMI and percentage of liver replacement, which could correlate with tumour aggressiveness. At last, the relation between BMI and tumour response seems linear, suggesting the correlation we observed is not exclusively imputable to a low response rate in the category of patients with the lowest BMI. Regarding TTP, the role of BMI was also significant but the type of relation could be different as in patients with the highest BMI, TTP seems to begin decreasing. A limit of these analyses was that BMI was recorded at the time of treatment, neglecting its evolution, whereas the latter is an important criterion of nutritional evaluation.

These results raise the interesting issue of BMI's role. Few studies focused on this issue. An elevated BMI is a risk factor for the occurrence of several cancers (Moertel *et al*, 1980; Calle *et al*, 2003), and is associated with a higher relapse rate after surgical treatment of colon, rectal and prostate cancer (Meyerhardt *et al*, 2003; Amling *et al*, 2004; Freedland *et al*, 2004; Meyerhardt *et al*, 2004). However, its impact on disease's natural history and response to treatment is less documented. A study about patients with hormonal refractory prostate cancer showed a positive correlation between BMI and survival (Halabi *et al*, 2005). It may be suggested that a lower BMI reflects a more aggressive disease, although this is difficult to define, and may refer to tumour extension, tumour kinetic, tumour biology or treatment sensitivity. The pathophysiological mechanisms involved are still unknown, and a role for adipokines might be hypothesised (Trayhurn and Wood, 2004). Other explanation could be advanced: chemotherapy dose was based on body surface area (mg m^−2^) such that patients with higher BMI may receive a larger total dose of chemotherapy. However, the chemotherapy was delivered via the hepatic artery. If it is assumed that (i) chemotherapy is selectively delivered to tumour vessels, (ii) there may be some first passage extraction and (iii) subsequent embolisation facilitates retention of drug in tumour, then the volume of distribution of drug is not the same as when given i.v. and is not a function of body surface area (except to the extent that liver size relates to this). Thus (assuming that tumour size is not correlated with BMI), the tumours of patients with higher BMI would be exposed to higher concentrations of chemotherapy. Assuming that chemotherapy has a dose–response effect, this may account for the relationship between BMI and objective response rate. This would also question the rationale of dosing according to body surface area in this context.

Two other factors were associated with tumour response: the use of streptozotocin, and the functionality of the tumour. However, these two factors did not correlate with TTP, and their predictive value must be taken cautiously. To account for this discrepancy, it can be hypothesised that our cohort was not large enough to show statistical significance for these factors in this peculiar model, or that the reason arterial phase enhancement and BMI are associated with TTP is not only their impact on tumour response but also a potential role on tumour kinetic. Regarding this issue, it must be stressed that MIB-1, which is a marker of cellular proliferation studied by immunohistochemistry and a well-documented prognosis factor (Chaudhry *et al*, 1992; Pelosi *et al*, 1996; Gentil Perret *et al*, 1998), should have been integrated in our two multivariate models according to univariate analyses, but, unfortunately, could not, as the number of missing data was too elevated. To clarify these questions, these factors, including MIB-1, should be studied, if possible in a larger population, from the time of diagnosis, and for BMI, followed-up.

As treatment assignment (streptozotocin *vs* doxorubicin) was not controlled nor randomised, it is not possible to conclude that streptozotocin is a more efficient drug. However, it suggests it is at least as efficient. Based on this, we see a potential advantage in using streptozotocin that is to save doxorubicin for subsequent use and chemotherapy, delaying the time we will face cardiac toxicity risk that sometimes hinders therapeutic strategy.

Efficacy of TACE does not seem to depend on the time it is performed early or late during the course of the disease (28% of patients had previously received chemotherapy), nor by the presence of extra-hepatic lesions. This must be discussed knowing half of the patients with extra-hepatic lesions had exclusively an unresected primary lesion or abdominal metastatic lymph nodes, and the others had a limited number of small lesions. The major prognostic role of liver metastases prompted TACE in these patients (Madeira *et al*, 1998; Gullo *et al*, 2003; Modlin *et al*, 2003; Plockinger *et al*, 2004).

Severe adverse events occurred after 17 TACE procedures (10%), but direct responsibility of treatment seems questionable for some of them (stroke, pulmonary embolism, coronary spastic angina). We did not observe any relation between baseline characteristics and the occurrence of adverse events. Risk factors for TACE-related adverse events have been described in studies focusing on hepatocellular carcinoma (Chung *et al*, 1996) (portal vein obstruction, biliary anastomosis or obstruction, compromised functional hepatic reserve). Except two patients who had a previous story of duodenopancreatectomy, none of our patients had such risk factors. A recent study suggested that replacement of more than 70% of liver by the tumour was associated with an increased morbidity from treatment (Roche *et al*, 2004). We did not confirm such findings, maybe in relation with the fact that operators perform a less complete embolisation, injecting less gelatin particles, in case of bulky disease.

Post embolisation syndrome was reported only after 47% of procedures in our series, whereas its frequency is usually reported to be higher (Ruszniewski *et al*, 1993; Therasse *et al*, 1993; Diaco *et al*, 1995; Dominguez *et al*, 2000; Gupta *et al*, 2003; Kress *et al*, 2003; O’Toole *et al*, 2003; Roche *et al*, 2003, 2004). We can account for this discrepancy by assuming that as this side effect is extremely frequent it was probably not systematically reported, so that we recorded it only when considered significant, that is, responsible for an extension of hospital stay or the use of major analgesics.

In conclusion, this study confirms previously reported results regarding TACE efficacy and tolerance for the treatment of liver metastases of endocrine tumours in a large series of patients. Our results suggest streptozotocin should be the drug of choice for TACE, as it allows saving anthracyclines for subsequent intravenous chemotherapy. We have identified several predictors of response to TACE, which should enable clinicians to better identify patients that will have a stronger benefit from treatment. In addition, the original association between BMI and response to treatment, if confirmed in subsequent studies, would raise interesting questions about its pathophysiological mechanisms. Although this study is one of the largest focusing on this topic, the number of patients included in the multivariate analysis remains limited, emphasising the need for larger, prospective multicentre studies in the future.

## Figures and Tables

**Table 1:** Baseline characteristics

		**Number of available data**
Median age, years (range)	57 (25–82)	67
Male gender, *n*	36	67
Body mass index, median (range)	23.7 (13.2–33)	64
		
*Performance status (ECOG), n*		67
0–1	62	
2	5	
Functioning tumour, *n*	38	67
		
*Primary lesion*		
Pancreatic location, *n*	19	67
Diameter (cm), median (range)	3 (0.8–16)	51
Resected, *n*	43	67
Extra-hepatic lesion(s), *n*	26	67
		
*Indication of treatment, n*		67
Progressive disease	41	
Aggressive disease	9	
Uncontrolled hormone-related symptoms	17	
Previous chemotherapy, *n*	19	67
Concomitant treatment with somatostatin analogs, *n*	25	67
MIB-1 (%), median (range)	3 (1–60)	34
		
*Radiological features of metastases*		
Longest diameter (cm), *n*	4 (1–14)	67
% of liver replaced by metastases, *n*		67
<25%	29	
>25 and <50%	14	
>50 and <75%	12	
>75%	12	
Arterial phase enhancement, *n*	42	61
Heterogeneous pattern, *n*	45	62
		
*Cytotoxic agent, n*		67
Streptozotocin	44	
Doxorubicin	23	

**Table 2:** Clinical, biological and tumour responses (%)

	**Clinical response (*n*=23)**	**Biological response (*n*=38)**	**Tumour response (*n*=67)**
Complete response	61	18	1
Partial response	30	47	36
Stable disease	4.5	32	36
Progressive disease	4.5	3	27

**Table 3:** Univariate analysis of baseline characteristics according to tumour response and time to progression (TTP)

	**Tumour response**			**TTP**		
	**Odds ratio**	**95% confidence interval**	***P***	**Hazard ratio**	**95% confidence interval**	***P***
Age^a^	1.01	(0.97; 1.06)	0.587	0.98	(0.95; 1.01)	0.189
Gender (F *vs* M)	1.44	(0.53; 3.91)	0.468	0.74	(0.40; 1.39)	0.347
Body mass index^a^	1.32	(0.99; 1.29)	0.069	0.86	(0.78; 0.95)	0.002
Pancreatic primary tumour	1.33	(0.45; 3.93)	0.610	1.11	(0.56; 2.17)	0.769
Functioning tumour	3.83	(1.27; 11.52)	0.016	0.72	(0.39; 1.33)	0.299
Primary lesion diameter^a^	0.77	(0.57; 1.03)	0.08	1.14	(1.02; 1.28)	0.019
Resected primary lesion	0.76	(0.27; 2.18)	0.615	1.4	(0.75; 2.62)	0.287
Extra-hepatic lesion(s)	0.83	(0.30; 2.30)	0.716	1.6	(0.87; 2.96)	0.131
Previous chemotherapy	0.97	(0.32; 2.92)	0.96	1.75	(0.92; 3.35)	0.091
Progressive or aggressive disease	0.56	(0.20; 1.64)	0.284	1.37	(0.69; 2.74)	0.371
Concomitant treatment with somatostatin analogues	0.91	(0.33; 2.55)	0.864	1.27	(0.69; 2.35)	0.447
MIB-1^a^	0.85	(0.67; 1.09)	0.197	1.04	(1.004; 1.069)	0.026
Hepatic lesion longest diameter^a^	0.99	(0.97; 1)	0.22	1.01	(0.99; 1.01)	0.314
Liver replacement >50%	0.57	(0.20; 1.66)	0.305	1.98	(1.06; 3.70)	0.033
Arterial phase enhancement	2.55	(0.72; 9.02)	0.146	0.58	(0.3; 1.14)	0.115
Hepatic lesion homogeneous pattern	0.99	(0.31; 3.18)	0.985	0.87	(0.41; 1.87)	0.728
Streptozotocin *vs* doxorubicin	2.83	(0.88; 9.04)	0.073	0.997	(0.49; 2.02)	0.993

a Quantitative variables.

**Table 4:** Side effects

**Side effects**	***n***
Post embolisation syndrome	79
Vagal reaction	8
Carcinoid crisis	5
Pulmonary embolism	3
Acute liver failure	3
Tumour lysis syndrome and acute renal failure	1
Stroke	1
Liver abscess	1
Coronary spastic angina	1
Grade III neutropaenia	1
Ascites	1
